# Identification of breast cancer candidate genes using gene co-expression and protein-protein interaction information

**DOI:** 10.18632/oncotarget.9132

**Published:** 2016-05-02

**Authors:** Zhenyu Yue, Hai-Tao Li, Yabing Yang, Sajid Hussain, Chun-Hou Zheng, Junfeng Xia, Yan Chen

**Affiliations:** ^1^ School of Life Sciences, Anhui University, Hefei, Anhui 230601, China; ^2^ Institute of Health Sciences, Anhui University, Hefei, Anhui 230601, China; ^3^ College of Electrical Engineering and Automation, Anhui University, Hefei, Anhui 230601, China

**Keywords:** breast cancer, gene co-expression, protein-protein interaction, subnetwork extraction algorithm, candidate gene

## Abstract

Breast cancer (BC) is one of the most common malignancies that could threaten female health. As the molecular mechanism of BC has not yet been completely discovered, identification of related genes of this disease is an important area of research that could provide new insights into gene function as well as potential treatment targets. Here we used subnetwork extraction algorithms to identify novel BC related genes based on the known BC genes (seed genes), gene co-expression profiles and protein-protein interaction network. We computationally predicted seven key genes (*EPHX2, GHRH, PPYR1, ALPP, KNG1, GSK3A* and *TRIT1*) as putative genes of BC. Further analysis shows that six of these have been reported as breast cancer associated genes, and one (*PPYR1*) as cancer associated gene. Lastly, we developed an expression signature using these seven key genes which significantly stratified 1660 BC patients according to relapse free survival (hazard ratio [HR], 0.55; 95% confidence interval [CI], 0.46–0.65; Logrank *p* = 5.5e−13). The 7-genes signature could be established as a useful predictor of disease prognosis in BC patients. Overall, the identified seven genes might be useful prognostic and predictive molecular markers to predict the clinical outcome of BC patients.

## INTRODUCTION

Breast cancer (BC) is the most common invasive cancer in females both in the developed and developing countries, with an estimated 234,190 new cases and 40,730 deaths expected in the United States in 2015 [1]. Risk factors for developing BC include obesity, lack of physical exercise, drinking alcohol, hormone replacement therapy during menopause, older age, first menstruation at early age, and so on. A familial history of BC also increases the risk of developing BC. Several mutations in *BRCA1*, *BRCA2* and *TP53* involve in a very high risk of BC. However, these mutations account for only a small portion of the total BC burden.

Most BCs are derived from the epithelial lining of the ducts or lobules. BC has been traditionally classified based on clinical and histopathologic characteristics such as histologic grade, stage of disease, and receptor status [2]. The classifications can affect the prognosis and the response to treatment. For example, poorly differentiated cancers often have the worst prognosis [3]. Since BC leads to high mortality, the early diagnosis especially the molecular diagnosis is particularly important for the therapy. Traditionally, treatment decisions have been based on tumor histology and three receptor biomarkers including ER (estrogen receptor 1), PR (progesterone receptor), and HER2 (erb-b2 receptor tyrosine kinase 2) [2]. Cancers that do not have any of these three receptor types are called triple-negative BC. They usually express receptors for other hormones [4]. Despite significant improvements in the treatment of BC, new therapies and treatment strategies are still needed.

So far, numerous genes have been found involved in breast tumorigenesis which can be acted as biomarkers for the early diagnosis and further clinical application. Although dozens of related genes have been found, they are insufficient to elucidate the tumorigenesis of BC unless more relevant genes being identified. Therefore, it is an extremely crucial task to discover novel candidate genes. It is time-consuming and cost-spending to discover disease related genes by experiment alone, because the search space is very large. Computational approach is an alternative method which can help investigators to cope with various biological problems, such as analyzing complex biological network [5–9] and identifying novel genes [10–11]. For example, Zhu et al. developed a robust geometric approach for modeling protein-protein interaction networks [8]. Huang et al. presented a model for predicting protein-protein interactions based on protein-protein correlation using least squares regression [9]. Deng et al. proposed a method to predict novel genes associated with cervical cancer through gene co-expression networks [10]. In addition, several studies have reported prognostic gene expression signatures for BC [12–14]. However, these studies have generally been limited by specific BC subtypes. Hence, development of a more robust molecular predictor that overcomes BC subtype variability is necessary as well as identification of novel genes.

In this study, a computational method was built to discover BC candidate genes based on known BC related genes retrieved from BCGD (the Breast Cancer Gene Database, http://www.tumor-gene.org/tgdf.html). After applying the subnetwork extraction algorithms with gene co-expression and protein-protein interaction (PPI) data, we obtained three networks containing the known BC related genes (denoted as seed genes) and the candidate genes (denoted as linker genes). Through comparing these three subnetworks, we found seven common candidate genes. Further analysis suggests that all of these seven genes are consistent with previous reports that they have relationship with BC or cancer. Using this 7-genes signature, we observed significant differences in relapse-free survival between low-risk and high-risk BC patients in Kaplan–Meier analyses. This analysis provides important insight into the subnetwork biomarkers associated with BC and the identified seven genes may be readily utilized for prognostication and risk-stratification of BC patients.

## RESULTS AND DISCUSSION

### Strategy for prediction of breast cancer candidate genes

**Figure 1:**
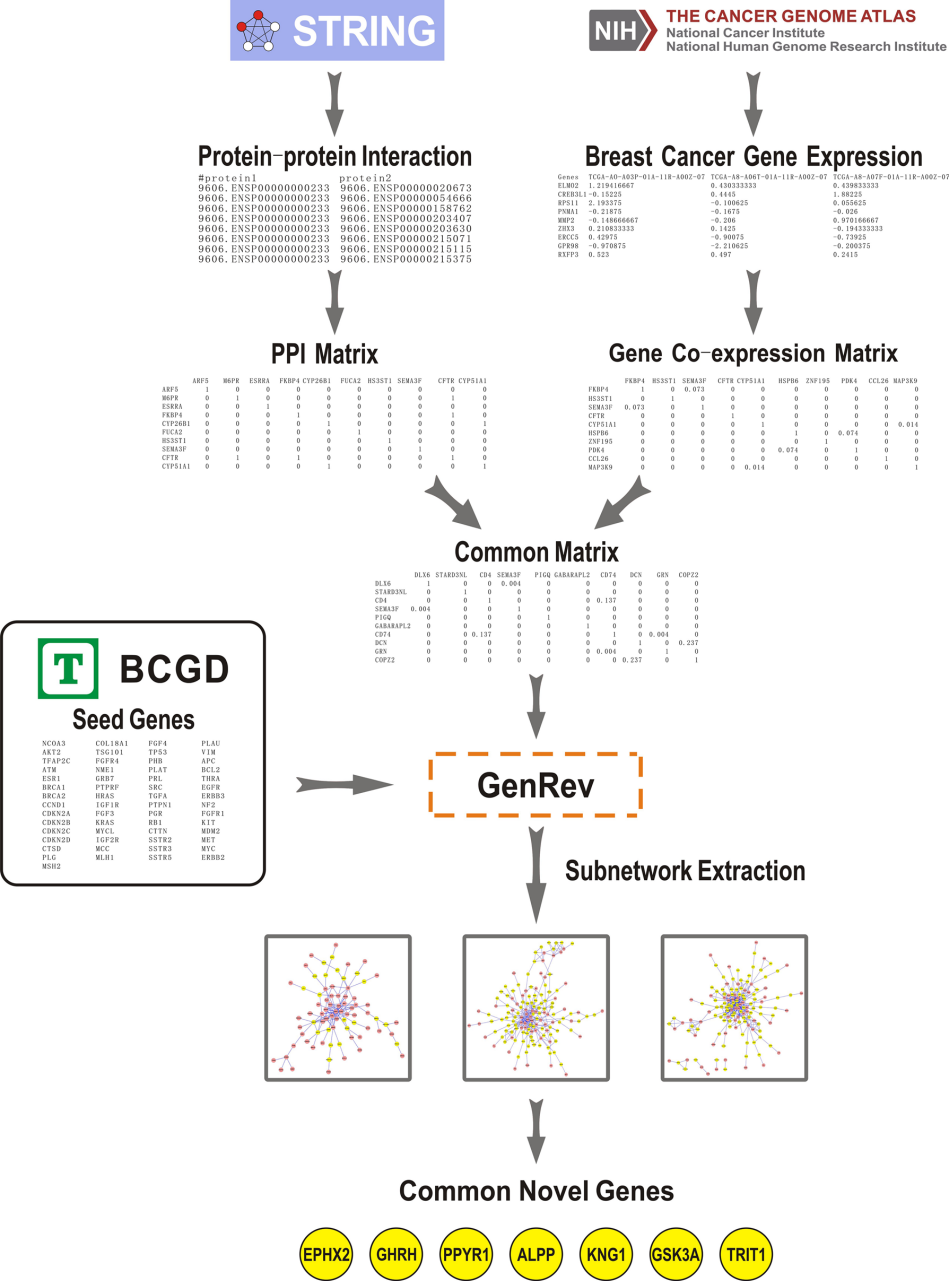
Summary workflow for identifying BC related candidate genes The approach was based on three steps: (1) We obtained a PPI network derived from STRING (Search Tool for the Retrieval of Interacting Genes/Proteins) and constructed a co-expression network using gene expression data from The Cancer Genome Atlas (TCGA). A common network was generated through comparing the two networks. (2) Seed genes and the common network were imported into GenRev which was used to extract three subnetworks with different extraction algorithms. (3) 7 common genes were found in the obtained subnetworks which were considered as key candidate genes related to BC.

Our goal is to use seed genes and a common biological network between gene co-expression and PPI networks with the subnetwork extraction algorithms to identify candidate genes that are related to the pathogenesis of BC. The method was based on three steps (Figure 1).

(1) We obtained a PPI network derived from STRING (Search Tool for the Retrieval of Interacting Genes/Proteins, http://string-db.org/), which is a database of known and predicted protein interactions [15]. Gene expression data of 531 BC tumor samples were collected from The Cancer Genome Atlas (TCGA, http://cancergenome.nih.gov) [16]. The co-expression network was constructed using R software package “WGCNA” [17]. Through comparing the PPI network and the gene co-expression network, a common biological network containing both co-expression and PPI information was generated. Table 1 illustrated the number of nodes and edges in PPI network, co-expression network and their common network, respectively.
(2) 57 seed genes were collected from BCGD, after removing 11 genes which are not included in the common network (Table 2). Seed genes and the common network were then imported into GenRev [18], a software developed to explore the functional relevance genes. We used the subnetwork extraction algorithms in GenRev for extracting three subnetworks (see more details in the Materials and Methods section).
(3) There were 7 common genes found in the three subnetworks, which were considered as key candidate genes related to BC. Then we analyzed these genes by retrieving existing literature and assessed the prognostic value of this 7-genes signature using the transcriptomic data by Kaplan Meier plotter, an online survival analysis software [19].

**Table 1:** Numbers of nodes and edges in PPI network, co-expression network and their common network were illustrated

	PPI Network	Co-expression Network	Common Network
Nodes	17460	17325	9534
Edges	4850628	2303648	148182

**Table 2:** Seed genes were collected from the breast cancer gene database (BCGD)

Gene Names			
NCOA3	COL18A1	FGF4	PLAU
AKT2	TSG101	TP53	VIM
TFAP2C	FGFR4	PHB	APC
ATM	NME1	PLAT	BCL2
ESR1	GRB7	PRL	THRA
BRCA1	PTPRF	SRC	EGFR
BRCA2	HRAS	TGFA	ERBB3
CCND1	IGF1R	PTPN1	NF2
CDKN2A	FGF3	PGR	FGFR1
CDKN2B	KRAS	RB1	KIT
CDKN2C	MYCL	CTTN	MDM2
CDKN2D	IGF2R	SSTR2	MET
CTSD	MCC	SSTR3	MYC
PLG	MLH1	SSTR5	ERBB2
MSH2			

A summary workflow for identifying genes critical to BC is illustrated in Figure 1.

### Subnetwork

As described in the Materials and Methods section, proteins in a PPI network or genes in a co-expression network may share some common or similar features. Therefore, after importing seed genes and the common network which represented both gene co-expression and PPI information into GenRev software, we searched the subnetworks connecting known BC related genes by three methods: not-weighted Klein-Ravi algorithm [20], not-weighted limited k-walk algorithm and edge-weighted limited k-walk algorithm [21].

**Figure 2:**
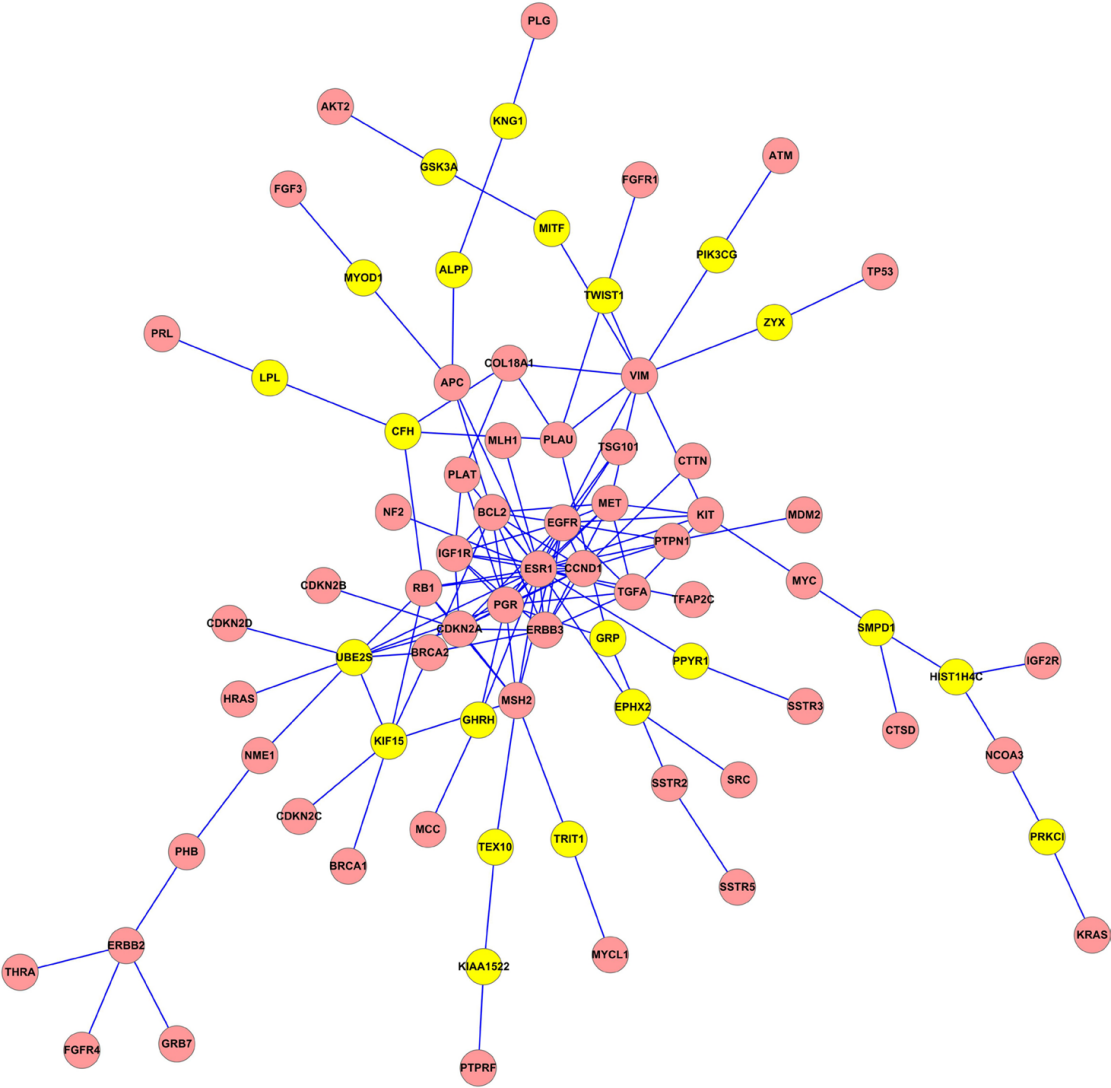
The subnetwork extracted by not-weighted Klein-Ravi algorithm

In this work, the linker genes were considered as the candidate genes related to BC that we found in the three obtained subnetworks. The whole linker genes were listed in Table S1. Numbers of edges, seeds and linkers in each network from different methods were showed in Table 3. As an example, Figure 2 illuminated the subnetwork extracted by not-weighted Klein-Ravi algorithm which contained 136 edges and 79 nodes (57 seed and 22 linker genes). The subnetworks extracted by not-weighted and edge-weighted limited k-walk algorithm were illuminated in Figures S1 and S2, respectively. Cytoscape software was used for visualizing subnetworks [22].

**Table 3:** Numbers of edges, seeds and linkers in the subnetworks obtained from different methods

Algorithm	Edges	Seeds	Linkers
Steiner	136	57	22
Kwalk (not-weighted)	268	57	87
Kwalk (edge-weighted)	348	57	83

Linkers refer to the novel genes. Steiner and Kwalk refer to the Klein-Ravi algorithm and the limited k-walk algorithm, respectively.

### Analysis of candidate genes

After comparing the genes from the three extracted subnetworks, we obtained 7 overlapping genes which might have a strong relationship with BC. Subsequent analysis indicates that six of these seven key genes (*EPHX2*, *GHRH*, *ALPP*, *KNG1*, *GSK3A* and *TRIT1*) have been shown as BC associated genes, and one (*PPYR1*) as cancer associated gene based on the existing literature. In the following we will discuss them one by one.

The *EPHX2* is the gene encodes a member of the epoxide hydrolase family which is found in both the cytosol and peroxisomes. It binds to specific epoxides and converts them to the corresponding dihydrodiols. Previous study has revealed that it can be targeted by tamoxifen in the treatment of BC [23] and downregulated by GW9662, a potent antagonist of PPARgamma that inhibits growth of breast tumor cells [24]. The protein encoded by *GHRH* is a member of the glucagon family of proteins. The encoded preproprotein is cleaved to generate somatoliberin, which acts to stimulate growth hormone release from the pituitary gland. Antagonists of this gene inhibit growth of various human cancers including BC. Splice variants of *GHRH* receptors could mediate the responses to *GHRH* and *GHRH* antagonists in BC through Ca^2+^-, cAMP- and PKC-dependent mechanisms [25]. The *PPYR1* gene encodes a member of neuropeptide Y family which is one of the most relevant neuropeptides related to tumor progression [26–27]. The protein encoded by *ALPP* is an alkaline phosphatase, a metalloenzyme that catalyzes the hydrolysis of phosphoric acid monoesters. Overexpression of this enzyme has been detected at the surface of various solid tumors [28]. Particularly, this gene is significantly higher expressed in the trastuzumab treated than in the untreated human HER2-amplified breast cancer cell line BT474 and can be an indicator for drug sensitivity [29]. The gene *KNG1* uses alternative splicing to generate two different proteins, low molecular weight kininogen and high molecular weight kininogen which is essential for blood coagulation. It has been reported that this gene is up-regulated by proteasome inhibitor in BC [30]. The protein encoded by *GSK3A* is a multifunctional Ser/Thr protein kinase that is implicated in the control of several regulatory proteins and transcription factors. It also plays a role in the WNT and PI3K signaling pathways which are closely associated with cancer. Induced expression of *PTEN* in *PTEN* deficient BC cells, was associated with a marked decrease in the basal phosphorylation of *GSK3A* and other downstream components of the PI3K signaling cascade, and then suppressed cell cycle progression [31]. The *TRIT1* encodes an isopentenyltransferase that is located to the mitochondrion and modifies tRNAs by adding a dimethylallyl group onto the adenine at position 37 which is considered a tumor suppressor [32]. The product of this enzyme, isopentenyladenosine significantly inhibited the BC cell lines MDAMB-361 and MCF7. The mechanism of tumor suppressor activity is associated with inhibition of cell proliferation, blocking DNA synthesis and morphological changes [33]. Overall, these seven key genes are shown to play a direct or indirect role in BC according to previous reports and merits further investigation with respect to their application against BC.

**Figure 3:**
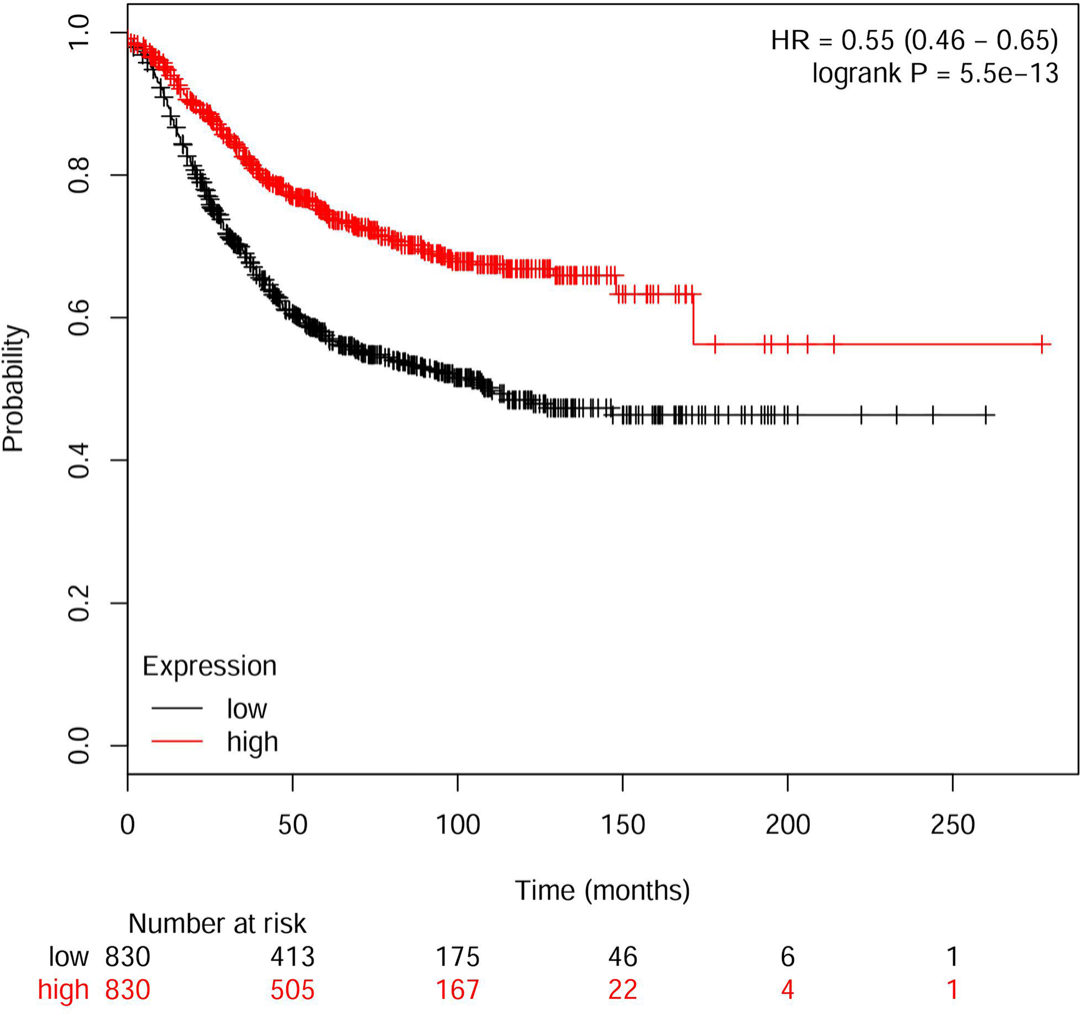
Kaplan–Meier plot of relapse free survival using the 7-genes signature

We then developed an expression signature consisting of the seven genes by the Kaplan Meier plotter online survival analysis software [19]. This 7-genes signature could significantly stratify 1660 BC patients according to relapse free survival (HR, 0.55; 95% CI, 0.46 − 0.65; Logrank *p* = 5.5e^−13^). As seen in Figure 3, the low-risk group had significantly better relapse free survival than the high-risk group. The 7-genes signature could be established as a useful predictor of disease prognosis in BC patients.

**Figure 4:**
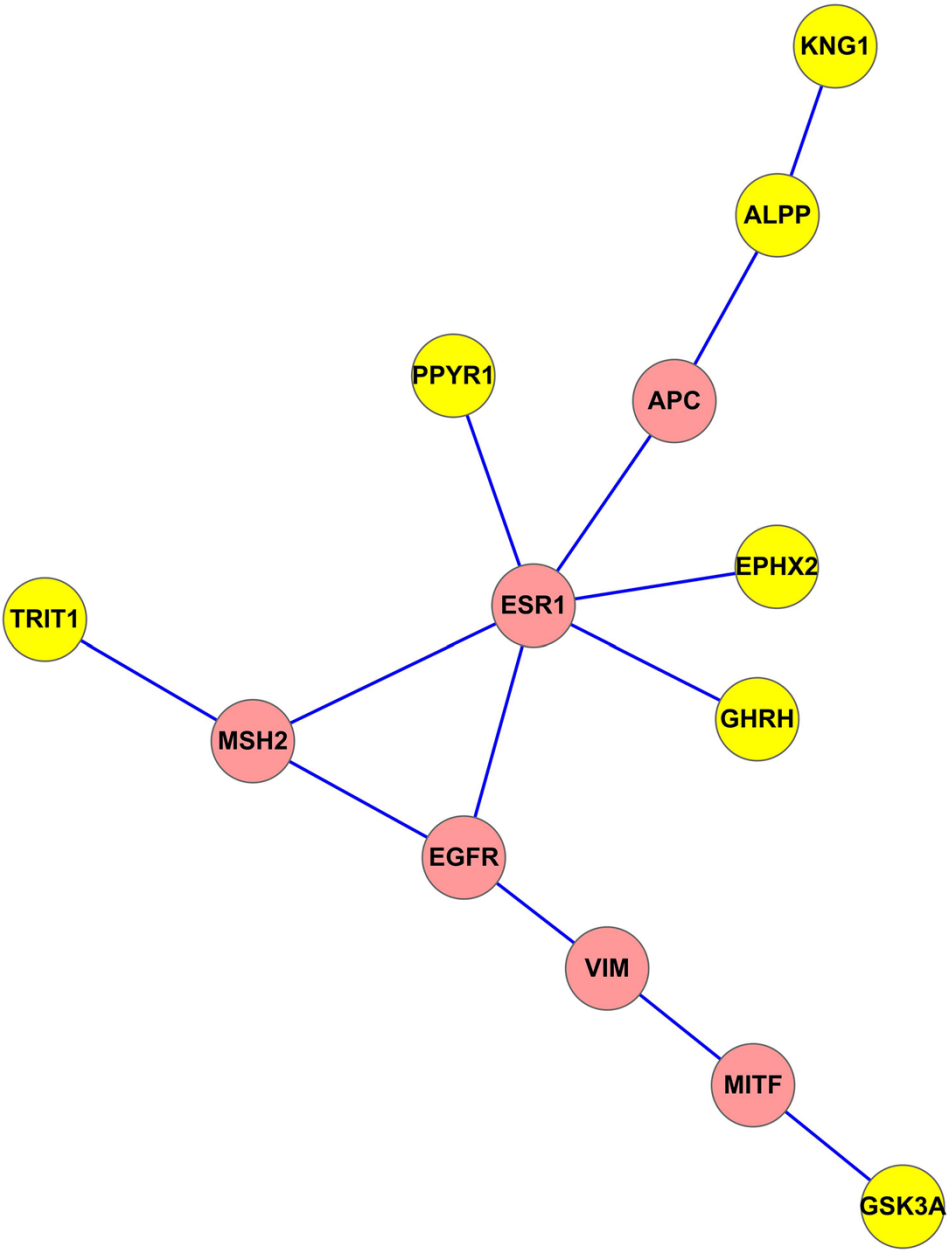
The smallest subnetwork connecting seven key genes derived from the common network

Finally, we identified the smallest subnetwork connecting seven key genes derived from the common network representing both PPI and gene co-expression information (Figure 4). The other six genes in this subnetwork are all cancer related: five of them are seed genes and one is *MITF* gene [34]. Paired *t*-test analysis showed statistically significant differences in these 13 gene expression levels between BC and normal samples (*p*-value = 0.049) using the gene expression data of 531 BC and 62 normal tissue samples from TCGA.

In summary, the identified seven candidate genes may help to reveal the underlying molecular mechanisms of BC and provide guidance orientation on possible personalized therapeutic regimen selection. It has not escaped our notice that the approach of this study may give a new insight to understand BC as well as other cancers. Besides the PPI and gene co-expression information used in this study, epigenetic and mutation level information also play an important role in BC. In the future work, we will identify driver mutations that have direct effect on a selective growth advantage of BC cells using these multidimensional data sets.

## MATERIALS AND METHODS

### Protein-protein interaction network

Protein-protein interaction (PPI) networks provide a lot of valuable information to understand cellular function and biological processes. Numerous studies have shown that proteins in the same interaction, that is, they are adjacent in the constructed PPI network, always have some common features [21, 35–38]. It can be further deduced that proteins in the subnetwork connecting known BC related genes are most likely to share similar biological functions as shown in many studies [39–40]. In this study, the PPI network was constructed based on the protein interaction information retrieved from STRING (Search Tool for the Retrieval of Interacting Genes/Proteins, http://string-db.org/) (9606.protein.links.v9.1) [15], a well-known online interaction repository which include direct (physical) and indirect (functional) associations.

### Gene co-expression network

Recently, gene co-expression network has emerged as a new tool for microarray analysis [41–42]. The transcript levels of two co-expressed genes rise and fall together across samples. It is showed that functionally related genes are frequently co-expressed constituting conserved transcription modules [43–44]. Here, gene expression microarray and RNA-Seq data of 531 BC tumor samples were collected from The Cancer Genome Atlas (TCGA, http://cancergenome.nih.gov) which aims to generate comprehensive, multi-dimensional maps of the key genomic changes in major types and subtypes of cancer [16]. Then a gene co-expression network was constructed using R software package “WGCNA” (Weighted correlation network analysis) which is a comprehensive collection of R functions for performing various kinds of weighted correlation network analysis [17]. The similarity was computed to evaluate the distance between each pair of genes using the function adjacency(). Pearson's correlation coefficient is used as the co-expression measure. The RNA-Seq data were transformed into log2 scale after adding a constant +1 as described in Meißner [45].

### Seed genes

BC related genes were collected from the Breast Cancer Gene Database (BCGD) which is a sub-database of Tumor Gene Family of Databases (TGFD) (http://www.tumor-gene.org/tgdf.html). After removing 11 gene which are absent in the common network representing both PPI and gene co-expression information, we obtained 57 genes as BC related genes (seed genes) with a high level of confidence (Table 2).

### Subnetwork extraction

Here, we used a method to discover candidate genes related to BC by constructing a common network contained both PPI and gene co-expression information. So far there are numerous methods that can be used to find the subnetworks. In this study, GenRev [18], a standalone and platform independent software for exploring the functional relevance genes, was used to identify subnetwork. The input files were comprised of seed genes and the common network. The Pearson's correlation coefficients in co-expression network were used as the weights of edges. Since the common network imported into GenRev contained only weights of edges but not weights of all nodes, we used the not-weighted Klein-Ravi algorithm, not-weighted and edge-weighted limited k-walk algorithm in GenRev [18]. As a result, GenRev was used mapped the genes to the common network and extracted three subnetworks. The subnetwork and the linker genes were visualized using Cytoscape software [22].

### Klein–Ravi algorithm

The Klein–Ravi algorithm is one of the algorithms in GenRev, which was proposed to solve the node-weighted Steiner tree problem [20]. The objective of the node-weighted Steiner tree problem was to find a subnetwork with a minimum score which connects all the seeds. The score of a subnetwork was estimated by the sum of the scores of its nodes. We can find more details from the original work [20].

### Limited k-walks algorithm

The limited k-walks algorithm is another algorithm in GenRev, which can run randomly in the network by using a Markov chain and build a relevant subnetwork connecting seed nodes [21]. The relevance of an edge and a node related to the seed genes is assessed by the expected times random walk passes starting from one seed to any of the others. By default, weights of all edges were equal to 1. The Pearson's correlation coefficients in co-expression network were set as weights of edges when the edge-weighted limited k-walk algorithm was used. More details are available in the original work [21].

### Kaplan Meier plotter

In this study, an online survival analysis software, Kaplan Meier plotter was used to assess prognosis value of the signature developed by the seven key candidate genes [19]. The Kaplan Meier plotter is capable of evaluating the effect of 54,675 genes on survival using 10,188 cancer samples using the log rank test to compare the survival curves. To analyze the prognostic value of 7 genes, the 3557 relapse-free survival samples were split into two groups according to various quantile expressions of the proposed biomarker. The two patient cohorts were compared by a Kaplan-Meier survival plot, and the hazard ratio with 95% confidence intervals and logrank *p-*value were calculated.

## SUPPLEMENTARY MATERIALS


