# Supplementary material for: Identification of breast cancer candidate genes using gene co-expression and protein-protein interaction information
Source: Oncotarget. 2016 May 2;7(24):36092–100. doi: 10.18632/oncotarget.9132 (PMC5094985; doi:10.18632/oncotarget.9132)
Supplement: Supplementary file 1 [file oncotarget-07-36092-s001.pdf]

Identification of breast cancer candidate genes using gene co-expression and protein-protein interaction information

Supplementary Materials

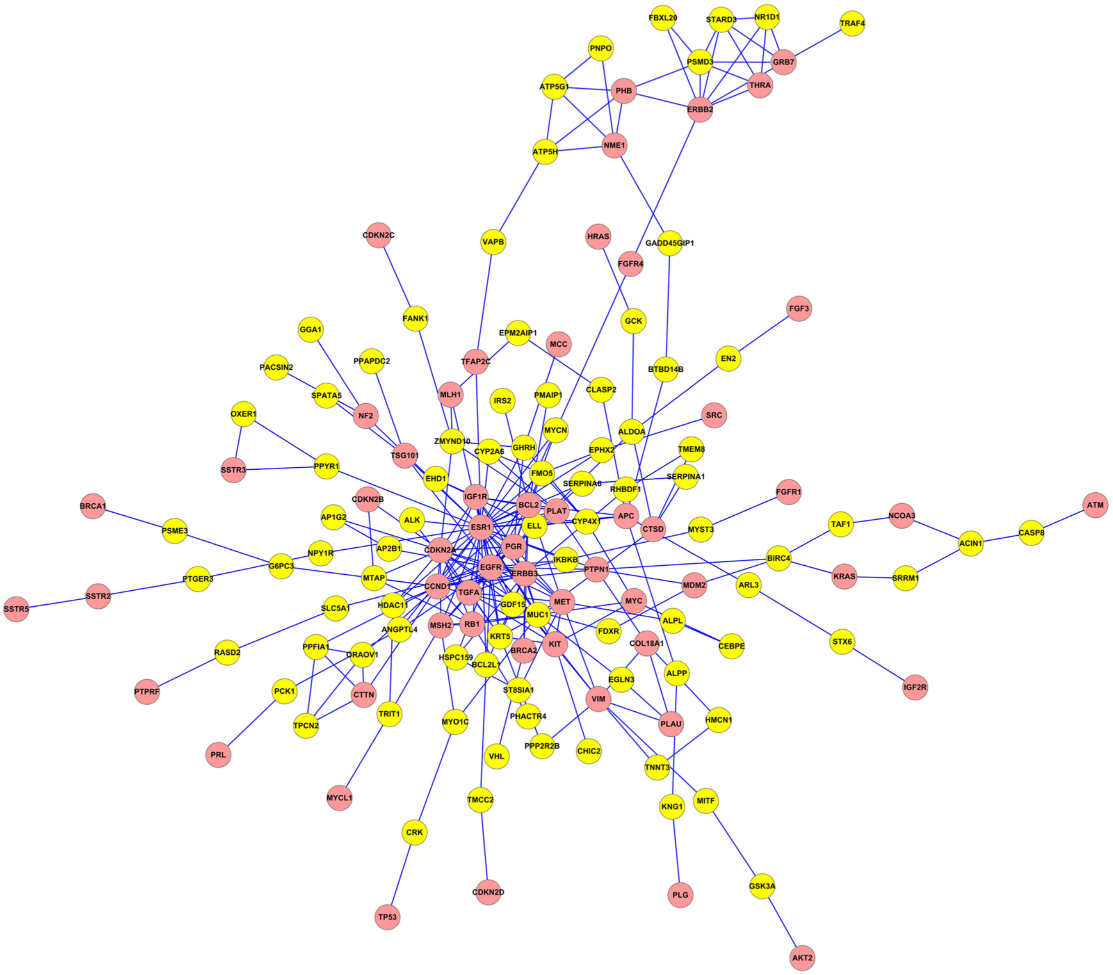

Supplementary Figure S1: The subnetwork extracted by not-weighted limited k-walk algorithm.

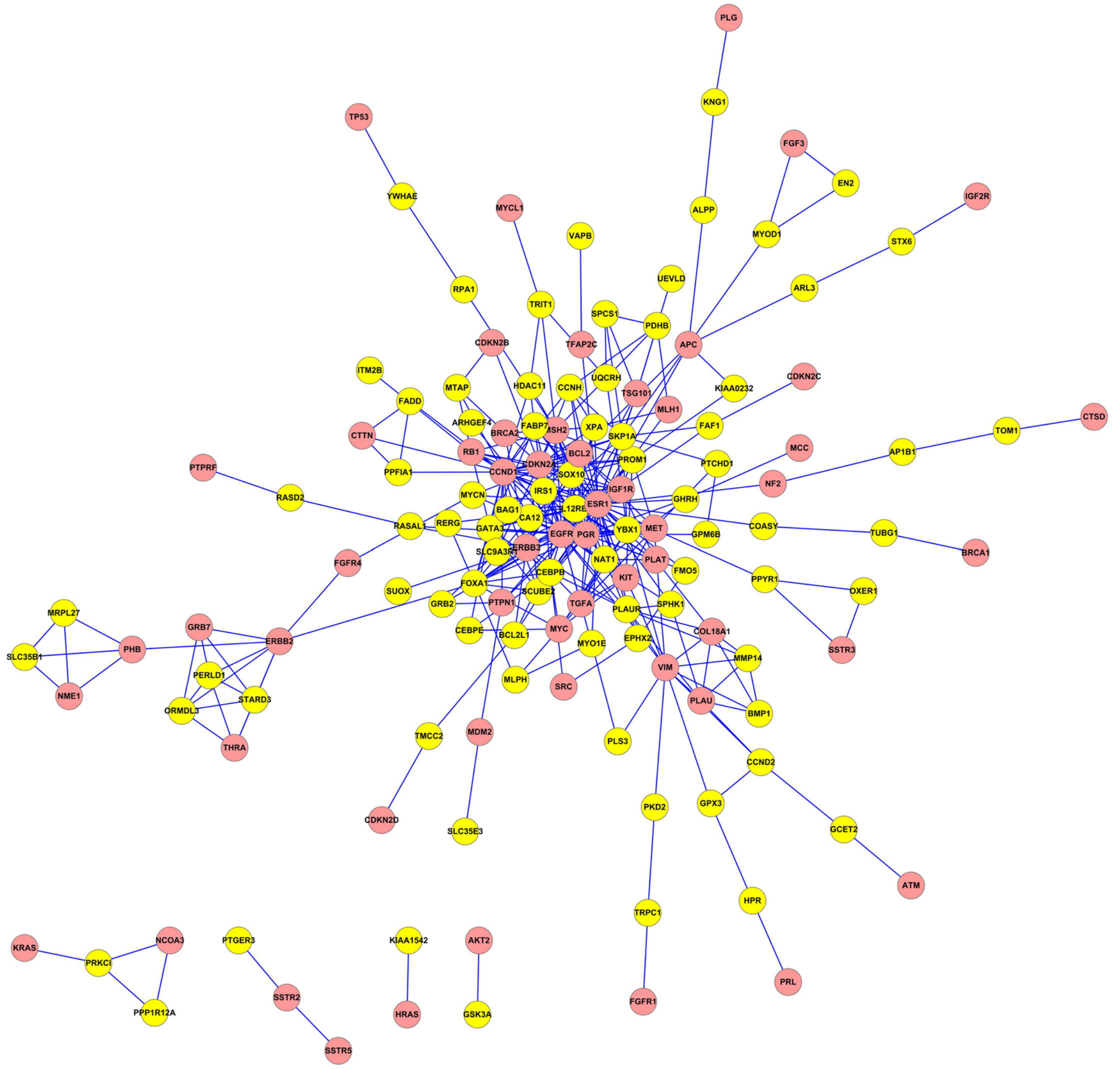

Supplementary Figure S2: The subnetwork extracted by edge-weighted limited k-walk algorithm.

Supplementary Table S1: Linker genes in the subnetworks obtained from different methods

Steiner	Kwalk(not-weighted)	Kwalk(edge-weighted)
HIST1H4C	BCL2L1	BCL2L1
EPHX2	ORAOV1	KIAA1542
GHRH	PCK1	BAG1
TEX10	PMAIP1	UQCRH
KIF15	PPAPDC2	GPM6B
SMPD1	IKBKB	RASAL1
MITF	FDXR	ARHGEF4
KIAA1522	FMO5	IL12RB2
ZYX	MUC1	FADD
MYOD1	ACIN1	FMO5
TWIST1	PPP2R2B	XPA
PIK3CG	CYP4X1	PROM1
GRP	HSPC159	GATA3
LPL	NPY1R	BMP1
PPYR1	GSK3A	CCND2
ALPP	GADD45GIP1	GSK3A
CFH	ANGPTL4	SKP1A
KNG1	PNPO	FOXA1
GSK3A	HDAC11	GRB2
UBE2S	RASD2	HDAC11
TRIT1	G6PC3	YBX1
PRKCI	CRK	MRPL27
	AP2B1	RASD2
	MYCN	MYCN
	TNNT3	SOX10
	EN2	EN2
	GDF15	PDHB
	MYST3	FAF1
	PTGER3	PTGER3
	GCK	PERLD1
	STARD3	STARD3
	EGLN3	SCUBE2
	TAF1	CCNH
	AP1G2	UEVLD
	ST8SIA1	SLC35B1
	RHBDF1	ITM2B
	FBXL20	GPX3
	EPHX2	ORMDL3
	GHRH	MMP14
	EHD1	EPHX2
	MYO1C	GHRH
	VAPB	MYO1E
	ALDOA	VAPB
	ATP5H	FABP7
	KRT5	TRPC1

	HMCN1	OXER1
	OXER1	COASY
	TMCC2	TMCC2
	ELL	GCET2
	GGA1	PPP1R12A
	BTBD14B	PLAUR
	TRIT1	ARL3
	ALK	NAT1
	TPCN2	PTCHD1
	SLC5A1	TRIT1
	KNG1	PKD2
	IRS2	KNG1
	STX6	IRS1
	PHACTR4	STX6
	ARL3	SPHK1
	BIRC4	SPCS1
	PSMD3	TOM1
	VHL	KIAA0232
	ZMYND10	CEBPB
	MITF	RERG
	NR1D1	MYOD1
	SRRM1	CEBPE
	SERPINA1	PPFIA1
	SERPINA6	RPA1
	PPFIA1	AP1B1
	ATP5G1	SLC35E3
	FANK1	SUOX
	PACSIN2	YWHAE
	CHIC2	PRKCI
	TRAF4	PLS3
	CLASP2	HPR
	PSME3	CA12
	CYP2A6	MTAP
	MTAP	PPYR1
	PPYR1	ALPP
	TMEM8	TUBG1
	ALPP	SLC9A3R1
	CEBPE	MLPH
	CASP8	
	EPM2AIP1	
	SPATA5	
	ALPL	

Linker genes refer to the novel candidate genes related to breast cancer.
